# Serum Concentrations of TIM-3, LAG-3, and PD-1 in Patients with Hemorrhagic Fever with Renal Syndrome

**DOI:** 10.3390/life14050551

**Published:** 2024-04-25

**Authors:** Željka Mačak Šafranko, Lana Jakopec, Karla Svaguša, Lidija Cvetko Krajinović, Domagoj Tomasović, ǈiljana Lukić, Alemka Markotić

**Affiliations:** 1 Research Unit, University Hospital for Infectious Diseases “Dr. Fran Mihaljevic”, 10000 Zagreb, Croatia; 2 Faculty of Medicine, University of Rijeka, 51000 Rijeka, Croatia; 3 Faculty of Medicine, Catholic University of Croatia, 10000 Zagreb, Croatia

**Keywords:** orthohantavirus, HFRS, PUUV, inhibitory receptors, immune regulations, TIM-3, LAG-3, PD-1

## Abstract

Hemorrhagic fever with renal syndrome (HFRS) is a rodent-borne disease widespread in Europe and Asia. HFRS is caused by negative-sensed single-stranded RNA orthohantaviruses transmitted to humans through inhaling aerosolized excreta of infected rodents. Symptoms of HFRS include acute kidney injury, thrombocytopenia, hemorrhages, and hypotension. The immune response raised against viral antigens plays an important role in the pathogenesis of HFRS. Inhibitory co-receptors are essential in regulating immune responses, mitigating immunopathogenesis, and reducing tissue damage. Our research showed an increased soluble form of inhibitory co-receptors TIM-3, LAG-3, and PD-1 in HFRS patients associated with disease severity. Our study aimed to investigate the impact of HFRS on the concentrations of soluble forms of inhibitory receptors TIM-3, LAG-3, and PD-1 in the patient’s serum and the potential correlation with key clinical parameters. Our study aimed to investigate the impact of HFRS on the concentrations of soluble forms of inhibitory receptors TIM-3, LAG-3, and PD-1 in the patient’s serum and their possible association with relevant clinical parameters. Using multiplex immunoassay, we found elevated levels of TIM-3, LAG-3, and PD-1 proteins in the serum of HFRS patients. Furthermore, increased levels were associated with creatinine, urea, lactate dehydrogenase concentrations, and platelet count. These findings suggest that these proteins play a role in regulating the immune response and disease progression.

## 1. Introduction

Hemorrhagic fever with renal syndrome (HFRS) is a significant public health concern as an emergent rodent-borne disease caused by negative-sense single-stranded RNA orthohantaviruses [1,2]. In Europe, including Croatia, there are various types of orthohantaviruses [3,4]. Puumala virus (PUUV) and Dobrava virus (DOBV) are pathogenic and have medical significance [5]. PUUV causes mild to moderately severe forms of the disease and is responsible for most recorded epidemics in Croatia. The occurrence of the disease is seasonal and correlates with the abundance of its natural host, the bank vole (*Myodes glareolus*), and mice of *Apodemus* spp. [3]. The primary pathologic features of HFRS are increased vascular permeability, low blood pressure, and acute kidney injury (AKI). In addition, patients with HFRS may develop disorders of the endocrine, nervous, cardiovascular, and respiratory systems, some of which can lead to multiorgan failure [6]. Biochemical parameters characteristic of HFRS are thrombocytopenia, elevated levels of creatinine and liver enzymes, proteinuria, hematuria, and leukocytosis [7].

The pathogenesis of HFRS is considered to be a complex process involving several factors, such as the pathologic effect of the virus and the immunological and genetic background of the host [8,9].

Inhibitory receptors are immune checkpoints that are expressed on immune cells to control and fine-tune the intensity and duration of the immune response. The immune response in acute viral infections is a well-regulated process that attempts to establish a balance between viral elimination and host protection. Inhibitory receptors play a crucial role by providing co-inhibitory signals to immune cells and dampening the immune response to prevent overreaction. Targeting these proteins influences the outcome of the immune response and disease progression and has been extensively studied in treating tumor diseases and chronic infections [10,11]. Repeated exposure to antigens in chronic infections can lead to T-lymphocytes becoming exhausted and unresponsive to cytokine stimulation. This can lead to a reduced proliferation potential as the expression of these receptors gradually increases [12]. Inhibitory receptors are commonly associated with adverse outcomes in tumors and chronic infections, and blocking their activity may have therapeutic benefits. However, the role of inhibitory receptors in acute infections is still unclear. Recent research has focused on the role of specific immune checkpoints, namely TIM-3 T-cell immunoglobulin and mucin-domain containing-3 (TIM-3), lymphocyte activation gene-3 (LAG-3) and programmed cell death protein 1 (PD-1), in modulating immune responses during viral infections. TIM-3 receptor is a transmembrane protein expressed on the surface of activated T-cells, regulatory T-cells, natural killer (NK) cells, macrophages, and dendritic cells (DC) [13,14]. Studies suggest reduced TIM-3 levels are associated with autoimmune diseases [15]. In contrast, TIM-3 is often overexpressed in chronic infections and cancer, resulting in a dampened immune response and hindered viral clearance, particularly in chronic infections [14,16]. The binding of TIM-3 to its ligand triggers cell death of Th1 cells and dampens Th1 immunity, thus preventing prolonged inflammation [17].

LAG-3 protein is primarily expressed in activated T-cells and NK cells. Its primary function is to inhibit their activation, which helps to suppress their cytotoxic activity and cytokine production during chronic infections and cancer [18,19]. LAG-3 also plays a vital role in promoting the differentiation of regulatory T-cells, which further contributes to immune tolerance. Mechanistically, LAG-3 exhibits a high affinity for binding to MHC class II molecules displayed on antigen-presenting cells (APCs), thereby modulating immune responses [20]. Although the LAG-3 protein does not directly impact cell survival and apoptosis, it exerts a regulatory effect on the cell cycle [21]. Shedding of the LAG-3 protein and its expression on the activated CD4+ T-cells strongly correlate with interferon-gamma (IFN-γ), a marker of activated T-cells [22]. LAG-3 expression is elevated on regulatory Tr1 lymphocytes, where it is associated with increased production of interleukin 10 (IL-10), an anti-inflammatory cytokine crucial for immune homeostasis [23]. The inhibitory protein PD-1 is expressed on various immune cells, including T- and B- lymphocytes, NK cells, activated monocytes, and DCs [24]. PD-1 interacts with its ligands PD-L1 and PD-L2 to inhibit T-cell activation and effector functions. Studies have shown that the expression of the PD-1 receptor on CD8+ lymphocytes affects their function and regulates the immune response during chronic infections such as HIV and HCV infections [25,26]. During acute HCV infection, the level of PD-1 receptor expression on CD8+ lymphocytes is higher compared to healthy individuals [26]. Similarly, during chronic HIV-1 infection, there is a higher level of PD-1 receptor expression in NK cells and CD8+ lymphocytes [27]. This checkpoint pathway is critical for maintaining immune tolerance and preventing excessive immune-mediated tissue damage [28,29].

This study investigated the involvement of the soluble form of inhibitory receptors TIM-3, LAG-3, and PD-1 in HFRS pathogenesis.

## 2. Materials and Methods

### 2.1. Test Subjects and Sample Collection

This study enrolled 28 individuals diagnosed with HFRS hospitalized at the University Hospital for Infectious Diseases “Dr. Fran Mihaljević” (UHID) in Zagreb, Croatia. The PUUV infection was consistently confirmed using a rapid immunohistochemistry test. Serum samples were collected from the patients during the acute phase of the disease upon admission to the hospital and from 10 sex- and age-matched healthy volunteers. Notably, all participants, patients, and healthy volunteers were residents of the Zagreb area, the capital of Croatia. Clinical and laboratory data were collected from the patient’s medical records of routine procedures. This study was conducted under the principles outlined in the Declaration of Helsinki, and each participant provided signed informed consent prior to participation, as approved by the UHID Ethics Committee. The isolated serum samples were maintained at a temperature of −80 °C.

### 2.2. Multiplex Immunoassay

Multiplex immunoassay panel ProcataPlex™ (Thermo Fisher Scientific, Waltham, MA, USA) was used to perform simultaneous quantification of TIM-3, LAG-3, and PD-1 in sera from HFRS patients and control subjects. The panel comprises antibody-conjugated microspheres, each specifically coated with antibodies targeting TIM-3, LAG-3, or PD-1. The protocol was performed according to the manufacturer’s instructions. In brief, aliquots of the serum samples were added to the wells of the panel containing the antibody-conjugated microspheres. The microspheres captured the target proteins (TIM-3, LAG-3, and PD-1) present in the serum through specific antibody–antigen interactions. After washing the unbound antibodies, a secondary antibody labeled with a fluorescent tag was added. The secondary antibody binds to the captured analyte and forms a sandwich complex. The fluorescence emitted by the microspheres, indicative of the amount of bound analyte, is then measured using a MAGPIX^®^ system (Luminex Corporation, Austin, TX, USA), which quantifies the analyte present based on fluorescence intensity. Fluorescence data obtained from the MAGPIX^®^ system were analyzed using appropriate software provided by the manufacturer.

Calibration curves generated using known standards were used to convert fluorescence intensity into quantitative measurements of TIM-3, LAG-3, and PD-1 concentrations in the serum samples.

### 2.3. Nested Polymerase Chain Reaction (PCR)

PUUV RNA was detected in sera from HFRS patients using the nested RT- PCR (reverse transcription polymerase chain reaction) method [30,31]. The total RNA from 0.20 mL of serum was extracted using the QIAamp blood mini kit (QIAGEN GmbH, Hilden, Germany). Reverse transcription (RT-PCR) was performed by transcribing five μL of RNA using the one-step access RT-PCR system (Promega, USA). The reverse transcription process proceeded for 60 min at 42 °C, followed by a denaturation step of 2 min at 94 °C. For the first PCR, primers PPT 334C (5′-TATGGIAATGTCCTTGATGT-3′) and PPT 986R (5′-GCACAIGCAAAIACCCA-3′) were used for DNA synthesis and amplification of the target DNA region. PCR conditions included denaturation of DNA strands, primer annealing, and extension. A 653 bp segment of the S protein was amplified. A nested PCR was then performed using primers PPT 376C (5′-CCIAGTGGICAIACAGC-3′) and PPT 716R (5’-AAICCIATIACICCCAT-3′) with HotStar Polymerase (Jena Bioscience GmbH, Jena, Germany). The reaction was run for 35 cycles at 94 °C for 30 s, 49 °C for 1 min, and 72 °C for 2 min. The PCR products were visualized on a 1% agarose gel. The detection of a 341 bp fragment confirmed the presence of PUUV RNA.

### 2.4. Statistical Analysis

Statistical analysis was conducted using the ggpubr 0.6.0 package in the R environment (version 4.2.3., R Core Team, Vienna, Austria) [32,33] and the open-source program JASP 0.17.2 [34]. Distribution normality was assessed graphically and with the Shapiro–Wilk test. Spearman’s correlation coefficient was used to determine the correlations between TIM-3, LAG-3, and PD-1 levels in the serum of HFRS patients and clinical parameters. *p*-values < 0.05 were considered statistically significant.

## 3. Results

### 3.1. Main Clinical Characteristics and Laboratory Findings of the Cohort Study Group

A total of 28 patients were enrolled in the study. Among these patients, 16 (57.1%) were aged between 20 and 39, while 9 (32.1%) were aged between 40 and 60. One patient was under 20 years old, and one was over 60. Twenty-one (75%) patients were male. The average disease duration was ten days, with a median of six days. All patients, 28 (100%), had fever, with a quarter experiencing hyperpyrexia (7 (25%)). Headache was another prominent symptom, impacting 27 (96.4%) patients. Blurred vision 11 (39.3%), nausea 17 (60.7%), and myalgia 21 (75%) were also frequently reported; 13 (46.6%) of the patients displayed hepatomegaly, while a smaller proportion, 6 (21.4%), exhibited splenomegaly. Hypotension was observed in 8 (28.6%) of the patients. Notably, proteinuria, a hallmark feature of HFRS, was present in 24 patients (85.7%) (Table 1).

Routine laboratory tests of HFRS patients showed elevated levels of urea (mean 8.3 mmol/L, standard deviation 5.3 mmol/L) and creatinine (mean 180.6 µmol/L, standard deviation 151.8 µmol/L) compared to normal ranges. Additionally, lactate dehydrogenase (LDH) activity was increased (mean 225.8 U/L, standard deviation 51.1 U/L). Consistent with the hallmark feature of HFRS, the minimum platelet count during hospitalization was below normal values (mean 72.4 × 10⁹/L, standard deviation 39.7 × 10⁹/L). While leukocyte counts varied within the reference range (minimum 7.1 × 10⁹/L, standard deviation 2.1; maximum 9.8 × 10⁹/L, standard deviation 2.3), the minimum red blood cell (RBC) count was slightly lower than normal (minimum mean 4.2 × 10⁹/L, standard deviation 0.5 × 10⁹/L; maximum mean 5, standard deviation 0.5). Urine output data demonstrated a range of oliguria (mean urine output, 1390.4 mL/day, standard deviation 852.4) (Table 2).

### 3.2. Increased Concentration of TIM-3, LAG-3, and PD-1 in HFRS Patients

**Figure 1:**
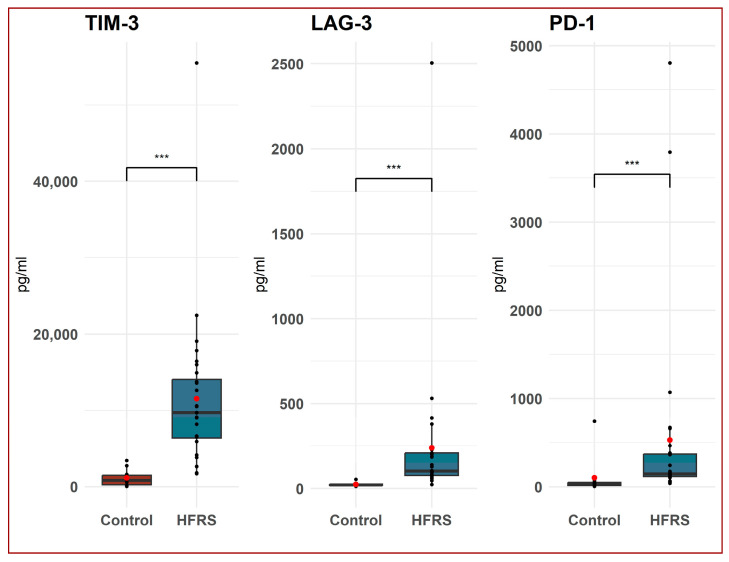
Comparison between TIM-3 T-cell immunoglobulin and mucin-domain containing-3 (TIM-3), lymphocyte activation gene-3 (LAG-3), and programmed cell death protein 1 (PD-1) protein concentrations in serum of patients with hemorrhagic fever with renal syndrome (HFRS) (*n* = 28) compared to healthy controls (*n* = 10). The data are presented in the form of a boxplot. A horizontal line represents the median of the data, and a red dot represents the mean. The box represents the interquartile range; outliers are shown as dots outside the whiskers. *p*-value calculated using the non-parametric Mann–Whitney test U test, *** *p*  <  0.001.

Our research showed that, during the acute phase of HFRS, the concentrations of soluble TIM-3, LAG-3, and PD-1 proteins in the serum of patients were significantly higher than those in the control group (*p* < 0.001). Specifically, the mean concentrations of these proteins in the patient group were 11,536.3, 239.3, and 530.4 pg/mL, respectively, with standard deviations of 10,210, 459.7, and 1098.24, respectively, while the mean concentrations in the control group were 1145.9, 24.3, and 102.8 pg/mL, respectively, with standard deviations of 1043.1, 11.4, and 226.2, respectively (Figure 1).

### 3.3. Differential Expression of LAG-3 and PD-1 in PUUV Negative Compared to PUUV-Positive HFRS Patients

**Figure 2:**
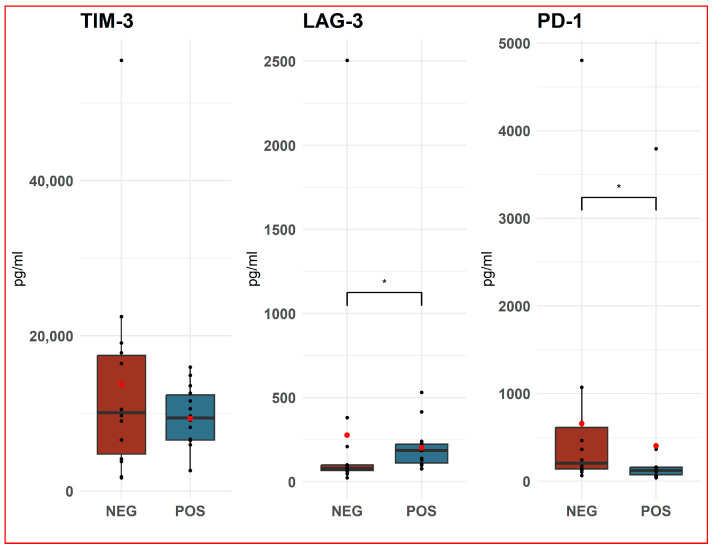
Comparison between TIM-3 T-cell immunoglobulin and mucin-domain containing-3 (TIM-3), lymphocyte activation gene-3 (LAG-3) and programmed cell death protein 1 (PD-1) protein concentrations (ng/mL) in serum of patients with hemorrhagic fever with renal syndrome (HFRS) positive to Puumala virus RNA (*n* = 14) compared to patients negative to Puumala RNA (*n* = 14). The data are presented in the form of a boxplot. A horizontal line represents the median of the data, and a red dot represents the mean. The box represents the interquartile range; outliers are shown as dots outside the whiskers. *p*-value calculated using the non-parametric Mann–Whitney test U test, * *p* < 0.05.

In samples that tested negative for PUUV RNA in serum, the concentration of TIM-3 (mean 13,739.6 pg/mL, standard deviation 13,729) was not significantly different compared to the positive group of HFRS patients (mean 9333 pg/mL, standard deviation 4190.9). The mean concentration of LAG-3 protein in the PUUV negative group (mean 277.0 pg/mL, standard deviation 647.3) was higher compared to PUUV-positive samples (mean 201.5 pg/mL, standard deviation 52.4, *p* < 0.05). However, it is important to approach these data with caution, as the median concentration was lower in negative samples (79.6) than in positive samples. The concentration of PD-1 protein in PUUV-negative samples was higher (mean 657.5 pg/mL, standard deviation 1227.5) than in positive samples (mean 403.3 pg/mL, standard deviation 981.6, *p* < 0.005) (Figure 2).

### 3.4. Correlation of TIM-3, LAG-3, and PD-1 Protein Concentration in Serum Samples with Clinical Parameters

**Figure 3:**
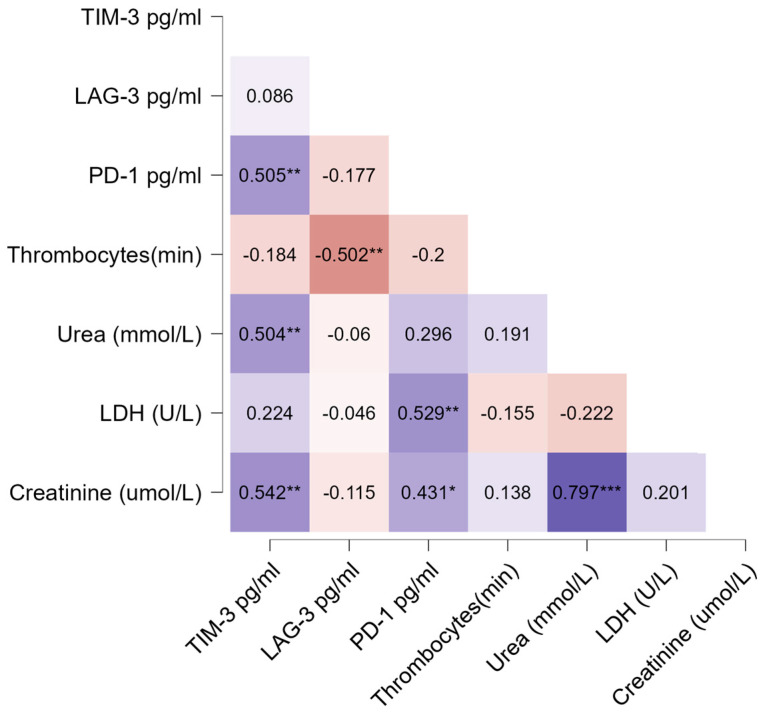
Heatmap visualization of Spearman’s correlation coefficients ρ of TIM-3, LAG-3, and PD-1 concentrations (pg/mL) in the early acute phase of HFRS and minimum platelet count (×10^9^/L) during hospitalization, Thrombocytes (min); concentration of urea, urea (mmol/L); concentration of lactate dehydrogenase, LDH (U/L); and concentration of creatinine, creatinine (µmol/L) on admission. Positive correlations (values above zero) are colored blue, and negative correlations (values below zero) are red. Statistical significance is pointed with asterisk * *p* < 0.05, ** *p* < 0.01, *** *p* < 0.001.

The heatmap visualization (Figure 3) shows the correlation between selected clinical parameters and protein concentrations. Data analysis using the Spearman correlation coefficient revealed a significant statistical correlation between the following: TIM-3 and PD-1 protein concentrations (ρ = 0.51, *p* < 0.01); TIM-3 protein and the maximum level of urea in the blood of patients during hospitalization, urea (max) (ρ = 0.46, *p* < 0.05); TIM-3 protein and creatinine levels (μmol/L) in the blood of patients on admission to the hospital (ρ = 0.54, *p* < 0.01); LAG-3 protein and number of thrombocytes (×10^9^/L) in blood on admission (ρ = −0.46, *p* < 0.05); protein PD-1 and creatinine level (μmol/l) on admission (ρ = 0.43, *p* < 0.05); and protein PD-1 and level of lactate dehydrogenase, LDH (U/L) in the blood of patients on admission (ρ = 0.53, *p* < 0.01).

## 4. Discussion

This study aimed to examine the influence of acute PUUV infection on the circulating levels of soluble forms of immune inhibitory receptors (TIM-3, LAG-3, and PD-1) in patients with HFRS. Our immunoassay results showed that the serum samples from patients with HFRS in the early acute stage exhibited higher concentrations of TIM-3, LAG-3, and PD-1 proteins compared to those of healthy controls.

Interestingly, TIM-3 protein level correlated with blood urea concentration, a marker of disease severity in HFRS. This finding is consistent with a study by Chen et al. (2022), which reported significantly higher concentrations of TIM-3 protein in the serum of patients with viral hepatitis compared to healthy controls. The study also emphasized the association of TIM-3 protein with the extent of liver damage and its potential influence on the pathogenesis of the disease [35]. Similarly, in chronic viral infections such as HIV and HBV, increased TIM-3 expression correlated with disease progression and viral load, while decreased frequencies of TIM-3 positive T-cells correlated with antiviral treatment and resolution of viral infection [36,37], suggesting that TIM-3 expression levels may be a prognostic indicator of disease progression in chronic viral infections [38]. In our research, we observed that the levels of TIM-3 were not associated with the presence of PUUV RNA in patients’ serum samples. However, we found that the mean concentration of LAG-3 was higher in samples that tested negative for PUUV than in those that tested positive. It is important to interpret these results with caution since the median value for LAG-3 was lower in the PUUV-positive samples.

The soluble form of LAG-3 has been shown to play a role in the maturation and migration of dendritic cells to secondary lymphoid organs, as well as in inhibiting the recruitment of antigen-presenting cells by suppressing the surface expression of CD1a or CD14 during the differentiation of monocytes into dendritic cells or macrophages [39]. We observed a correlation between the high levels of LAG-3 protein in the serum of HFRS patients and a decrease in platelet count. Thrombocytopenia, a well-known indicator of the severity of HFRS and acute renal failure [40], is believed to be influenced by the immune response of the host. Our findings indicate that LAG-3 plays a role in this process.

PD-1 receptor has a significantly more important role in maintaining self-tolerance and preventing self-toxicity compared to TIM-3 and LAG-2 [38]. Our research showed a significant increase in the concentration of PD-1 protein in the serum of HFRS patients. In addition, upregulation of PD-1 in hantavirus infection on CD4+ lymphocytes in the early stage of the disease was previously reported [41]. Furthermore, we found elevated levels of PD-1 in samples negative for PUUV, suggesting an association of PD-1 with viral load. It is known that orthohantaviruses, in addition to endothelial cells, can infect monocytes and dendritic cells [42]. These cells, along with lymphocytes, are potential sources of soluble forms of the inhibitory receptors measured by immunoassay. Orthohantavirus-infected endothelial and dendritic cells can enhance the expression of PD-L1 and PD-L2 ligands, a suggested mechanism of immune evasion. This phenomenon has also been observed in other viral infections [43]. 

Upregulation of PD-1 was found to be associated with higher viral load in mouse models of acute lymphocytic choriomeningitis virus (LCMV) infection, and its blockade helped overcome the infection [44]. However, in human Ebola virus infection, only PD-1 co-expressed with another inhibitory receptor (CTLA-4) on CD8+ T-cells, not PD-1 alone, correlated with viral load [45]. Although our results differ from those of previous studies, it is important to note that we only detected the viral genome in the samples we collected and did not measure viral load. In HFRS infection, viral RNA is detectable in the early stages of the disease, but we did not find a significant difference in the concentration of PD-1 depending on the onset of symptoms. We also found a positive correlation between PD-1 and creatinine levels in the blood of the patients, indicating a possible link between PD-1 expression and renal function (reflected by creatinine levels). 

We also found a positive correlation between PD-1 and TIM-3, which is consistent with several studies that demonstrated the co-expression of PD-1 and TIM-3 on T-cells, particularly CD8+ T-cells, indicating terminal T-cell exhaustion [46,47]. The observed correlations between TIM-3, PD-1, creatinine, and urea levels emphasize the complex interplay between immune regulation and renal function, indicating their involvement in this process.

## 5. Conclusions

In summary, our research demonstrated the complex relationship between acute PUUV infection and the levels of the soluble immune inhibitory receptors TIM-3, LAG-3, and PD-1 in patients with HFRS. We observed increased concentrations of these proteins in the serum of HFRS patients in the early acute phase compared to healthy controls. Notably, TIM-3 levels correlated with markers of disease severity, such as blood urea concentration, suggesting its potential as a prognostic indicator. Furthermore, we found interesting associations between LAG-3 and platelet count, suggesting its role in thrombocytopenia, a hallmark of HFRS severity. In addition, our results emphasized the importance of upregulation of PD-1, with implications for viral load and renal function, as evidenced by its correlation with creatinine levels. The observed positive correlation between PD-1 and TIM-3 highlights the complexity of immune regulation in HFRS and possibly indicates terminal T-cell exhaustion. Our study emphasizes the intricate interplay between immune regulation, viral infection, and renal function in the context of HFRS, providing valuable insights for further research and potential therapeutic interventions.

## Figures and Tables

**Table 1:** Clinical parameters of the HFRS patients.

Clinical Findings	Number of Patients (%)
Fever	28 (100.0)
Hyperpyrexia (>40 °C)	7 (25.0)
Headache	27 (96.4)
Myalgia	21 (75.0)
Blurred vision	11 (39.3)
Nausea	17 (60.7)
Hepatomegaly	13 (46.6)
Splenomegaly	6 (21.4)
Hypotension	8 (28.6)
Proteinuria	24 (85.7)

**Table 2:** Significant Laboratory findings of HFRS patients.

Laboratory Findings	Mean	Std. Deviation	Minimum	Maximum
Urea (mmol/L)	8.3	5.3	2.6	23.7
Creatinine (umol/L)	180.6	151.8	63	669
LDH (U/L)	225.8	51.1	154	340
Thrombocytes (minimum) × 10^9^/L	72.4	39.7	19	192
Leukocytes (minimum) × 10^9^/L	7.1	2.1	1.5	12.3
Leukocytes (maximum) × 10^9^/L	9.8	2.3	5.1	14.2
Erythrocytes (minimum) × 10^9^/L	4.2	0.5	2.4	4.9
Erythrocytes (maximum) × 10^9^/L	5	0.5	3.9	6.3
Urine output (minimum) mL/day	1390.4	852.4	50	3800

## Data Availability

The data presented in this study are available at the request of the corresponding author.
